# BRCA1/p220 loss triggers BRCA1-IRIS overexpression via *mRNA* stabilization in breast cancer cells

**DOI:** 10.18632/oncotarget.462

**Published:** 2012-03-19

**Authors:** Yoshiko Shimizu, Nicole Mullins, Zannel Blanchard, Wael M. ElShamy

**Affiliations:** ^1^ Cancer Institute and Department of Biochemistry, University of Mississippi Medical Center, Jackson, MS.

**Keywords:** BRCA1/p220, BRCA1-IRIS, breast cancer, RNA stability, survival, invasion/metastasis

## Abstract

*BRCA1/p220*-assocaited and triple negative/basal-like (TN/BL) tumors are aggressive and incurable breast cancer diseases that share among other features the no/low BRCA1/p220 expression. Here we show that BRCA1/p220 silencing in normal human mammary epithelial (HME) cells reduces expression of two RNA-destabilizing proteins, namely AUF1 and pCBP2, both proteins bind and destabilize BRCA1-IRIS mRNA. BRCA1-IRIS overexpression in HME cells triggers expression of several TN/BL markers, e.g., cytokeratins 5 and 17, p-cadherin, EGFR and cyclin E as well as expression and activation of the pro-survival proteins; AKT and survivin. BRCA1-IRIS silencing in the TN/BL cell line, SUM149 or restoration of BRCA1/p220 expression in the mutant cell line, HCC1937 reduced expression of TN/BL markers, AKT, survivin, and induced cell death. Collectively, we propose that BRCA1/p220 loss of expression or function triggers BRCA1-IRIS overexpression through a post-transcriptional mechanism, which in turn promotes formation of aggressive and invasive breast tumors by inducing expression of TN/BL and survival proteins.

## INTRODUCTION

Women with *BRCA1/p220* mutations are predisposed to early-onset breast cancer [1,2]. Although triple negative/basal-like (TN/BL) are sporadic tumors, they share many phenotypical, immunohistochemical, clinical and molecular characteristics with *BRCA1/p220*-mutant cancers [3,4]. Loss of BRCA1/p220 tumor suppression function often leads to profound increase in genomic instability [5,6], likely due to lack in DNA damage repair [7], in cell-cycle checkpoints activation [8] or in ubiquitylation-mediated degradation of proliferation (e.g., estrogen receptor [ER]) or survival (e.g., AKT) proteins [9-11].

Apoptosis evasion allows further transforming mutations to accumulate in cancer cells and increase the possibility of disease progression and/or resistance to therapy [12,13]. About 50% of breast cancers carry dysfunctional p53 [14], and thus fail to arrest the cell cycle when damaged becoming chemo-drug resistant [14]. Similarly, ~40% of breast cancers show increase AKT kinase activity [15] and are apoptosis and chemo-drug resistant [16,17].

Transcription upregulation, enhanced mRNA stabilization or suppression of protein degradation can all lead to increase in gene expression [18]. The rate of decay of certain mRNAs is regulated by the interaction of sequence-specific *trans*-acting, mRNA destabilizing proteins (reviewed in [19]), such as the poly(U)-binding factor (*aka* hnRNPD/AUF1, see [20,21]) and the poly(rC)-binding proteins (*aka* hnRNPE/pCBP1-4, see [22]) or mRNA stabilizing proteins, such as HuR (*aka* ELAVL1, see [23]) with *cis*-acting AU- or C-rich elements (ARE) in the 3`-UTR of these mRNAs. In human, ~10% of the genes mostly oncogenes are regulated by this post-transcriptional mechanism [24-27]. Not surprisingly, several of these destabilizing proteins are downregulated in cancers [28,29].

BRCA1-IRIS is a recently identified, 1399 residue *BRCA1/p220* locus proto-oncogene [30] made from the first 11 exons and 34-amino acid encoded by BRCA1/p220 intron 11 (for details see [30]). BRCA1-IRIS overexpression inhibits geminin function, thus promoting DNA replication [30], triggers cyclin D1 expression, thus promoting cell proliferation [31,32] and prevents p53 and/or p38MAPK activation or enhances AKT and survivin expression/activation, thus increases cell tolerance to cell-/geno-toxic stimuli [33,34].

Here, we show that BRCA1-IRIS overexpression in breast tumor cells is, at least partially, BRCA1/p220-dependent. BRCA1/p220 silencing in HME cells downregulated expression of the RNA destabilizing proteins, AUF1 and pCBP2, that bind to *BRCA1-IRIS mRNA* 3`-UTR and destabilize it. BRCA1-IRIS overexpression in HME cells triggered expression of the TN/BL markers, cytokeratins 5 and 17 (CK5 and 17), p-cadherin (CDH3), EGFR and cyclin E [35] as well as expression and activation of the survival factors, AKT and survivin. BRCA1-IRIS silencing or BRCA1/p220 overexpression in BRCA1/p220-mutant or TN/BL cancer cell lines reduced expression of these TN/BL markers, AKT and survivin and induced cell death. Our data show that BRCA1/p220 loss of expression or function generates aggressive breast cancer cells, in part, by upregulating BRCA1-IRIS expression, implying that chemotherapeutic targeting of BRCA1-IRIS could be pursued for breast cancer patients with *BRCA1/p220*-associated or TN/BL diseases.

## RESULTS

### BRCA1-IRIS overexpression in BRCA1/p220 none-/low-expressing breast cancer cells

**Figure 1:**
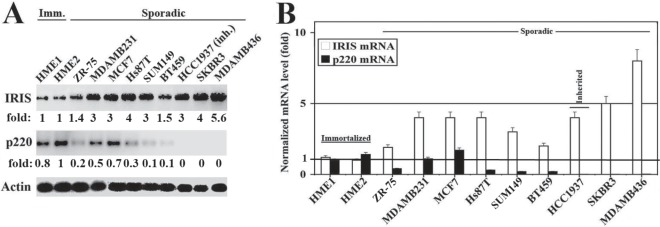
Expression of BRCA1-IRIS and BRCA1/p220 in breast cancers cell lines Western blot (A) and RT/PCR (B) analysis of BRCA1-IRIS and BRCA1/p220 in immortalized normal HME cell lines (HME1 and HME2), sporadic and one inherited (HCC1937) breast cancer cell lines. The RNA levels in (B) are normalized to the levels of GAPDH found in each cell line.

We showed earlier an inverse correlation between BRCA1-IRIS and BRCA1/p220 expressions in breast cancer cell lines [30]. Here, we confirmed that by analyzing proteins and RNAs isolated from several exponentially growing breast cancer cell lines and 2 normal HME cell lines. As expected western blot analysis using mouse monoclonal antibodies [30,36] and real time RT/qPCR, respectively, showed that in normal HME cell lines expressing high levels of BRCA1/p220 protein (Figure 1A) and *mRNA* (Figure 1B), BRCA1-IRIS protein (Figure 1A) and *mRNA* (Figure 1B) levels were significantly lower. In contrast, in sporadic or *BRCA1/p220*-mutant (e.g., HCC1937), low or none BRCA1/p220-expressing cell lines, respectively (see figure 1A and 1B), BRCA1-IRIS mRNA and protein expression significantly increased (Figure 1A and 1B).

### BRCA1/p220 does not affect BRCA1-IRIS protein stability

**Figure 2:**
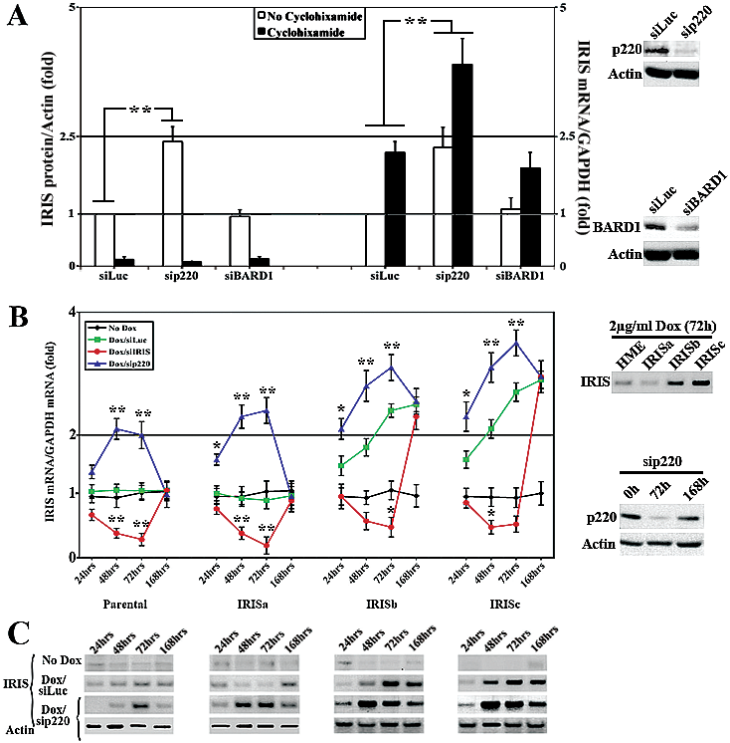
BRCA1/p220 silencing triggers BRCA1-IRIS expression in HME cells (A) Western blot (right) or RT/qPCR (left) analysis of the fold induction in *BRCA1-IRIS* protein normalized to actin or mRNA normalized to *GAPDH* mRNA, respectively in HME cells silenced (for 72h) from control (Luc), BRCA1/p220 and BARD1 and treated or not with cycloheximide during the last 24h. Data represent the means ± SD from triplicate, done three independent times, whereas ** is a p≤0.01. Far right panels show the effects of BRCA1/p220 (upper panels) and BARD1 (lower panels) siRNA on the expression of their cognate protein in HME cells. RT/qPCR analysis (B) or western analysis (C) of BRCA1-IRIS *mRNA* or protein, respectively in parental, uninducible IRISa and inducible IRISb and IRISc HME cell lines following control or BRCA1/p220 silencing for 24, 48, 72 or 168h. Data in (B) represent the means ± SD from triplicate, done three independent times, whereas * is a p≤0.05 and ** is a p≤0.01. Right panels in (B) show analysis for BRCA1-IRIS overexpression in the different inducible cell lines (upper panels), and the effect of BRCA1/p220 siRNA on the expression of BRCA1/p220 protein at 0, 72 and 168h (lower panels).

To understand the underlying molecular mechanism behind this inverse relationship, we considered three mutually exclusive scenarios. BRCA1/p220 could directly or indirectly; a) suppress *BRCA1-IRIS* gene transcription, b) decrease *BRCA1-IRIS* mRNA stability, or c) trigger BRCA1-IRIS protein degradation (BRCA1/p220 forms an E3 ligase with BARD1, see [9,10]). To distinguish between these possibilities, BRCA1/p220 or BARD1 were silenced in HME cells for 72h (see Figure 2A, far right panels) and cells were exposed to 10μM of cycloheximide (protein synthesis inhibitor) during the last 24h.

The levels of BRCA1-IRIS and actin proteins were measured using western blot on proteins isolated from these cells using sonication. All data were normalized to actin protein level in siLuc/no cycloheximide treated cells, which was taken as 1 (Figure 2A, left). As expected BRCA1-IRIS protein level decreased following cycloheximide treatment in control-, BARD1- and BRCA1/p220-silenced cells (Figure 2A, left). In the absence of cycloheximide, however, BRCA1-IRIS protein level was higher in BRCA1/p220-silenced cells, compared to control and BARD1-silenced cells (Figure 2A, left). Moreover, the levels of *BRCA1-IRIS* and *GAPDH* mRNAs was measured using real-time RT/qPCR on RNAs isolated from these cells. All data were normalized to *GAPDH* mRNA level in siLuc/no cycloheximide treated cells, which was taken as 1 (Figure 1A, right). *BRCA1-IRIS* mRNA level increased in control and BARD1-silenced cells following cycloheximide treatment only (Figure 1A, right), whereas in BRCA1/p220-silenced cells before and after cycloheximide treatment (Figure 1A, right). These data argue against an effect of BRCA1/p220 and/or BRAD1 on the stability of BRCA1-IRIS protein. In fact, previously we were unable to detect any interaction between BRCA1-IRIS protein and BRCA1/p220 or BARD1 proteins *in vitro* or *in vivo* (see [30]).

### BRCA1/p220 destabilizes BRCA1-IRIS mRNA

Next, we studied whether BRCA1/p220 affects *BRCA1-IRIS* mRNA stability (known to be controlled by elements in the 3`-UTRs of mRNAs). A BRCA1-IRIS cDNA that includes the entire 3`-UTR of *BRCA1-IRIS* (see [30]) was cloned in a doxycycline (Dox) inducible mammalian expression vector, infected in HME cells and one uninducible (IRISa) and two inducible (IRISb and c) clones were selected to study further (Figure 2B, right upper panel). We reasoned that since BRCA1-IRIS is expressed in these cells from an exogenous promoter, they should be a good system to explore whether BRCA1/p220 affects BRCA1-IRIS expression by a transcriptional or post-transcriptional mechanism. Thus parental, IRISa, b and c were grown in the absence or presence of Dox (2μg/ml), in the presence of Dox but cells were transfected with BRCA1-IRIS or BRCA1/p220 siRNAs. RNAs and proteins (using sonication) were isolated at 24, 48, 72 or 168h post-siRNA transfection and the expression of BRCA1-IRIS mRNA and protein in each treatment was measured using real time RT/PCR (Figure 2B, left) and western blot (Figure 2C), respectively.

In the absence of Dox the 4 cell lines expressed normal levels of BRCA1-IRIS mRNA (black lines in Figure 2B, left) and protein (Figure 2C). Dox induced *BRCA1-IRIS* mRNA (compare green to black lines in Figure 2B, left) and protein (Figure 2C) expression in IRISb and c and not parental or IRISa starting at 24h. BRCA1-IRIS silencing decreased *BRCA1-IRIS* mRNA (see red lines in Figure 2B, left) and protein (Figure 2C) in all cell lines (although less pronounced in induced IRISb and IRISc). The expression returned to pre-siRNA transfection levels at 168h (see red lines in Figure 2B, left and 2C). BRCA1/p220 silencing (Figure 2B, right lower panels), however, increased *BRCA1-IRIS* mRNA (blue lines in Figure 2B, left) and protein (Figure 2C) levels in all cell lines starting at 24h. These effects also disappeared at 168h post-siRNA transfection (blue lines in Figure 2B, left and 2C) when the effect of BRCA1/p220 siRNA disappeared (see Figure 2B, right lower panels).

To ascertain that these effects are dependent on the 3`-UTR of *BRCA1-IRIS* mRNA and not an artifact from the plasmid 5`-UTR (known inducer of mRNA translation), *BRCA1-IRIS +* 3`-UTR cDNA was cloned into several other mammalian expression vectors. Transient transfection of any of the plasmids into HME cells with BRCA1/p220 siRNA led to stabilization of the *BRCA1-IRIS* mRNA (not shown). We thus concluded that BRCA1/p220 loss stabilizes *BRCA1-IRIS* mRNA through an effect on its 3`-UTR.

### Identification of BRCA1/p220 induced trans-acting proteins that de-stabilize BRCA1-IRIS mRNA

In an attempt to identify whether BRCA1/p220 has an effect on mRNA stability, we searched recently performed gene expression microarray data comparing BRCA1/p220-expressing to BRCA1/p220-silenced HME cells for 3`-UTR binding and destabilizing proteins. Using this approach we found that the expressions of the mRNA 3`-ITR binding and destabilizing proteins, AUF-1 and pCBP2 (see introduction) were significantly decreased in BRCA1/p220-silenced HME cells (data not shown).

**Figure 3:**
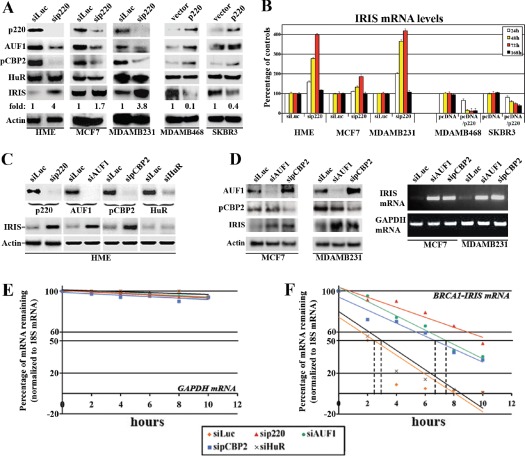
BRCA1/p220 controls BRCA1-IRIS mRNA stability (A) Expression of the indicated proteins in BRCA1/p220-silenced HME, MCF7 and MDAMB231 cells (left) or BRCA1/p220 overexpressing MDAMB468 and SKBR3 cells (right). (B) Expression of *BRCA1-IRIS mRNA* in BRCA1/p220-silenced HME, MCF7 and MDAMB231 cells (left) or BRCA1/p220 overexpressing MDAMB468 and SKBR3 cells (right). (C) Expression of the indicated proteins in BRCA1/p220-, AUF1-, pCBP2- or HuR-silenced HME cells (upper panel) and the expression of BRCA1-IRIS in these cells (lower panels). (D) Expression of the indicated proteins (72h, left) or *BRCA1-IRIS mRNA* (at 25 PCR cycle, at 72h) in control-, AUF1-, or pCBP2-silenced MCF7 or MDAMB231 cells. Stability (t_1/2_) of *GAPDH* (E) or *BRCA1-IRIS* (F) *mRNAs* as detected using real-time RT-qPCR analysis in BRCA1/p220-, AUF1-, pCBP2- or HuR-silenced HME cells treated with Actinomycin D (Act D) during the last 10h. Data are normalized to the levels of 18S rRNA in each experiment and are represented as a percentage of the mRNA levels measured at time 0 (before Act D addition) or 2, 4, 6, 8 and 10h after Act D addition using a semi-logarithmic scale. Data are presented as the means ± SD from triplicates, done three independent times (in all cases p≤0.001 compared to sicontrol cells).

To confirm that, we silenced BRCA1/p220 for 24-168h in normal HME cells, MCF7 (estrogen receptor-positive, ER^+^) or MDAMB231 (ER^−^) breast cancer cell lines (both express detectable levels of BRCA1/p220 protein, see Figure 1A and 3A, left). We also transiently overexpressed (for 24-168h) BRCA1/p220 in two none-/low-BRCA1/p220 expressing breast cancer cell lines, MDAMB468 (TN/BL) and SKBR3 (Her2^+^) (see Figure 1A).

Compared to control treated cells, BRCA1/p220 silencing (72h) significantly reduced AUF1 and pCBP2 but not HuR protein levels in HME, MCF7 and MDAMB231 cells, and its overexpression (48h) in MDAMB468 and SKBR3 cells significantly enhanced AUF1 and pCBP2 but not HuR protein levels (see Figure 3A, right). In contrast, BRCA1/p220-silencing increased the level of BRCA1-IRIS protein in HME, MCF7 and MDAMB231 to different degrees (see Figure 3A, left), whereas its overexpression significantly decreased BRCA1-IRIS protein expression in MDAMB468 and SKBR3 cells (Figure 3A, right). This was also confirmed at the mRNA level. Indeed in real-time RT/PCR and after normalization to the level of *GAPDH* mRNA in each cell line, we found that BRCA1/p220 silencing increased *BRCA1-IRIS* mRNA in HME, MCF7 and MDAMB231 cells starting at 24h (Figure 3B, left) until 72h, but returned to control levels at 168h (Figure 3B, left). BRCA1/p220 overexpression, on the other hand, decreased the level of *BRCA1-IRIS* mRNA in MDAMB468 and SKBR3 cells starting at 24h and thereafter (Figure 3B, right).

To directly assess the effect of AUF1 and pCBP2 on BRCA1-IRIS expression, they and BRCA1/p220 were separately silenced in HME cells (72h, Figure 3C, upper panels), MCF7 or MDAMB231 (72h, Figure 3D, left). AUF1 or pCBP2 and not HuR (control) silencing like BRCA1/p220 silencing significantly increased the level of BRCA1-IRIS protein in HME (Figure 3C, lower panels), MCF7 and MDAMB231 (Figure 3D, left) cells. Moreover, AUF1- or pCBP2-silencing increased *BRCA1-IRIS* mRNA levels in MCF7 and MDAMB231 cells (shown at 25 PCR cycle at 72h, Figure 3D, right). These data confirm that expression of BRCA1-IRIS in normal and cancer cell lines is, at least partially, BRCA1/p220/AUF1 and pCBP2-dependent.

### BRCA1/p220 effect on BRCA1-IRIS is post-transcriptional and not transcriptional

To rule out any effect of BRCA1/p220 on BRCA1-IRIS gene transcription and to directly assess the effect of BRCA1/p220, AUF1 or pCBP2 on *BRCA1-IRIS* mRNA stability, *BRCA1-IRIS* mRNA half-life (t_1/2_) was analyzed in control-, BRCA1/p220-, AUF1-, pCBP2- or HuR-silenced (72h) HME cells following exposure to the *de novo* transcription inhibitor actinomycin D (Act D) during the last 10h. Total RNAs were collected at 2, 4, 6, 8 and 10h after Act D treatment and analyzed by real-time RT/qPCR for *BRCA1-IRIS* and *GAPDH* expression.

After normalization to the level of *18S* rRNA, the none-target and stable *GAPDH* mRNA level remained unchanged following all siRNA transfections (Figure 3E). *BRCA1-IRIS* mRNA t_1/2_, on the other hand, was 2.5-3h in control and HuR-silenced cells (Figure 3F), increased to >10h in BRCA1/p220-silenced (Figure 3F) and to >6h in AUF1- or pCBP2-silenced HME cells (Figure 3F). These data demonstrate that BRCA1/p220 does not affect *BRCA1-IRIS* gene transcription, but instead destabilizes *BRCA1-IRIS* mRNA.

### Identification of destabilizing cis-acting elements in BRCA1-IRIS mRNA 3`-UTR

**Figure 4:**
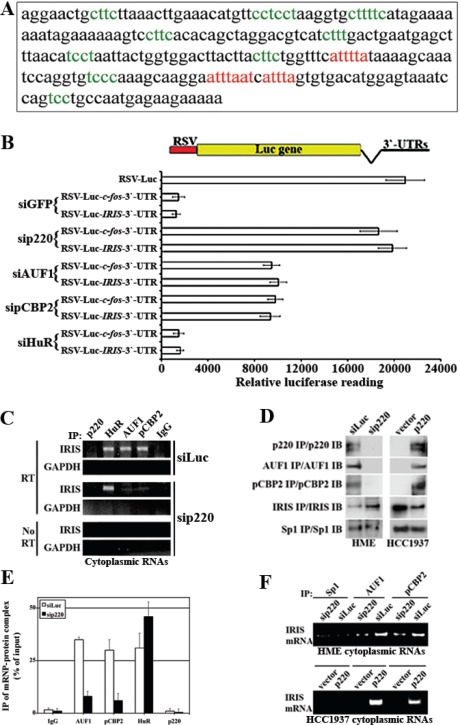
Identification and analysis of AREs motifs in BRCA1-IRIS 3`-UTR region (A) The 3`-UTR region of *BRCA1-IRIS* (i.e. part of BRCA1/p220 intron 11) where putative AUF-1 (red sequences) and pCBP2 (green sequences) binding sites are shown. (B) Plasmids carrying *BRCA1-IRIS* or *c-fos* 3`UTRs downstream of the luciferase gene in RSV-plasmid were transfecetd in HME with siGFP, siBRCA1/p220, siAUF1, sipCBP2 or siHuR and the luciferase activity expressed from these different plasmids was measured using luminometer. Data are represented as means ± SD from triplicates done three independent times (in all cases p≤0.01 compared to RSV-Luc alone). (C) Representative PCR analyses of the binding of *BRCA1-IRIS mRNA* to AUF1, pCBP2, HuR in untreated HME cells cytoplamsic proteins (polysomes), while binding only to HuR in BRCA1/p220-silenced HME cells. (D) Immunoprecipitation of the indicated proteins in BRCA1/p220-silenced HME cells (left), or from BRCA1/p220-reconstituted HCC1937 cells (right). (E) Immunoprecipitated mRNP-protein complex as percentage of input using AUF1, pCBP2, HuR and BRCA1/p220 antibodies and IgG from cells transfected with control or BRCA1/p220 siRNA. (F) Representative PCR analyses of the binding of AUF1 and pCBP2 to *BRCA1-IRIS* mRNA in BRCA1/p220-silenced HME cells (upper) or BRCA1/p220-overexpressing HCC1937 (lower) cytoplasmic proteins (polysomes).

Indeed, *in silico* search of *BRCA1-IRIS* 3`-UTR showed the presence of several *cis*-acting putative ARE consensus binding elements for AUF1 (class II AU rich binding motifs, see red in Figure 4A) and for pCBP2 (C-rich binding motifs, see green in Figure 4A), suggesting that the 3`-UTR destabilizes *BRCA1-IRIS* mRNA, *in vivo*. To experimentally confirm that, the entire *BRCA1-IRIS* 3`-UTR was cloned downstream of the luciferase coding region in the RSV-Luc plasmid (see atop of Figure 4B). The 3`-UTR of *c-fos* (known ARE containing) was also cloned downstream of the luciferase coding region in the same plasmid to be used as positive control. Backbone plasmid transfection in HME cells produced high-level basal luciferase expression (Figure 4B). Introducing *BRCA1-IRIS* or *c-fos* 3`-UTR downstream of the luciferase gene in this plasmid abolished that expression when co-transfected in HME cells with siGFP and siHuR (negative controls, Figure 4B), but not when co-transfected with BRCA1/p220, AUF1 or pCBP2 siRNA (Figure 4B). Taken together these data suggest that like *c-fos* 3`-UTR, *BRCA1-IRIS* 3`-UTR carry AUF1 and pCBP2 binding and mRNA destabilizing motif(s).

To measure endogenous association between these *trans*-acting factors and the *cis*-acting elements in *BRCA1-IRIS* 3`-UTR, cytoplasmic proteins (polysomes) were collected 72h after control- or BRCA1/p220-silencing in HME cells in conditions that maintain RNA stability and were processed for immunoprecipitation with BRCA1/p220, AUF1, pCBP2, HuR or IgG (negative control) antibodies. All immunoprecipitated samples were DNaseI digested to ensure they were free from any genomic contaminations before they were RT/PCR interrogated for *BRCA1-IRIS* mRNA. *GAPDH* mRNA (none target) was not immunoprecipitated by any antibody (Figure 4C), and no PCR amplification was detected when no reverse transcriptase (RT) was added to the reactions (Figure 4C). In siLuc-transfected cells, AUF1, pCBP2, HuR and not IgG or BRCA1/p220 antibodies co-immunoprecipitate *BRCA1-IRIS* mRNA (Figure 4C). This association was abolished when *BRCA1/p220* was silenced (Figure 4C).

Finally, to confirm that these antibodies immunoprecipitate their cognate proteins, HME cells were transfected with BRCA1/p220 siRNA or the BRCA1/p220 mutant cell line; HCC1937 was infected with wild type BRCA1/p220 expressing virus. Sp1 (negative control) antibody immunoprecipitated similar amounts of Sp1 from control and BRCA1/p220-silenced HME cells (Figure 4D, left) as well as vector and BRCA1/p220 infected HCC1937 cells (Figure 4D, right). Higher AUF1 and pCBP2 were immunoprecipitated from control- than BRCA1/p220-silenced HME cells (Figure 4D, left), and increased amount of BRCA1-IRIS was immunoprecipitated from BRCA1/p220- compared to control-silenced HME cells (Figure 4D, left). Furthermore, BRCA1/p220, AUF1 and pCBP2 were immunoprecipitated from BRCA1/p220- and not vector-infected HCC1937 cells (Figure 4D, right) and increased amount of BRCA1-IRIS was immunoprecipitated from vector- compared to BRCA1/p220-infected HCC1937 cells (Figure 4D, right).

The amounts of *BRCA1-IRIS* mRNA immunoprecipitated from control or BRCA1/p220 silenced HME cells by IgG, AUF1, pCBP2, HuR and BRCA1/p220 antibodies were compared to the total *BRCA1-IRIS* mRNA in polysome extracts using RT/qPCR. While equal amount of *BRCA1-IRIS* mRNA was immunoprecipitated with AUF1, pCBP2 and HuR antibodies from control treated cells (~30-35% of total BRCA1-IRIS polysomic mRNA see siLuc in Figure 4E), the amount immunoprecipitated by AUF1 and pCBP2 dropped to <10%, while the amount immunoprecipitated by HuR antibody increased to ~50% in BRCA1/p220-silenced cells (Figure 4E). In line with that, from polysome extracts, Sp1 antibody immunoprecipitated no *BRCA1-IRIS* mRNA from controls, BRCA1/p220-silenced HME cells or BRCA1/p220-overexpressing HCC1937 cells (Figure 4F). BRCA1/p220 silencing in HME cells decreased, whereas BRCA1/p220 overexpression in HCC1937 cells increased the level of *BRCA1-IRIS* mRNA immunoprecipitated by AUF1 or pCBP2 antibody (Figure 4F). These data show that *BRCA1-IRIS mRNA* is a target for the mRNA de/stabilizing factors AUF1, pCBP2 and HuR in normal and breast cancer cells, and that AUF1, pCBP2 and not HuR expression is BRCA1/p220-dependent in these cells.

### Loss of BRCA1/p220 enhances TN/BL phenotype via BRCA1-IRIS overexpression

**Figure 5:**
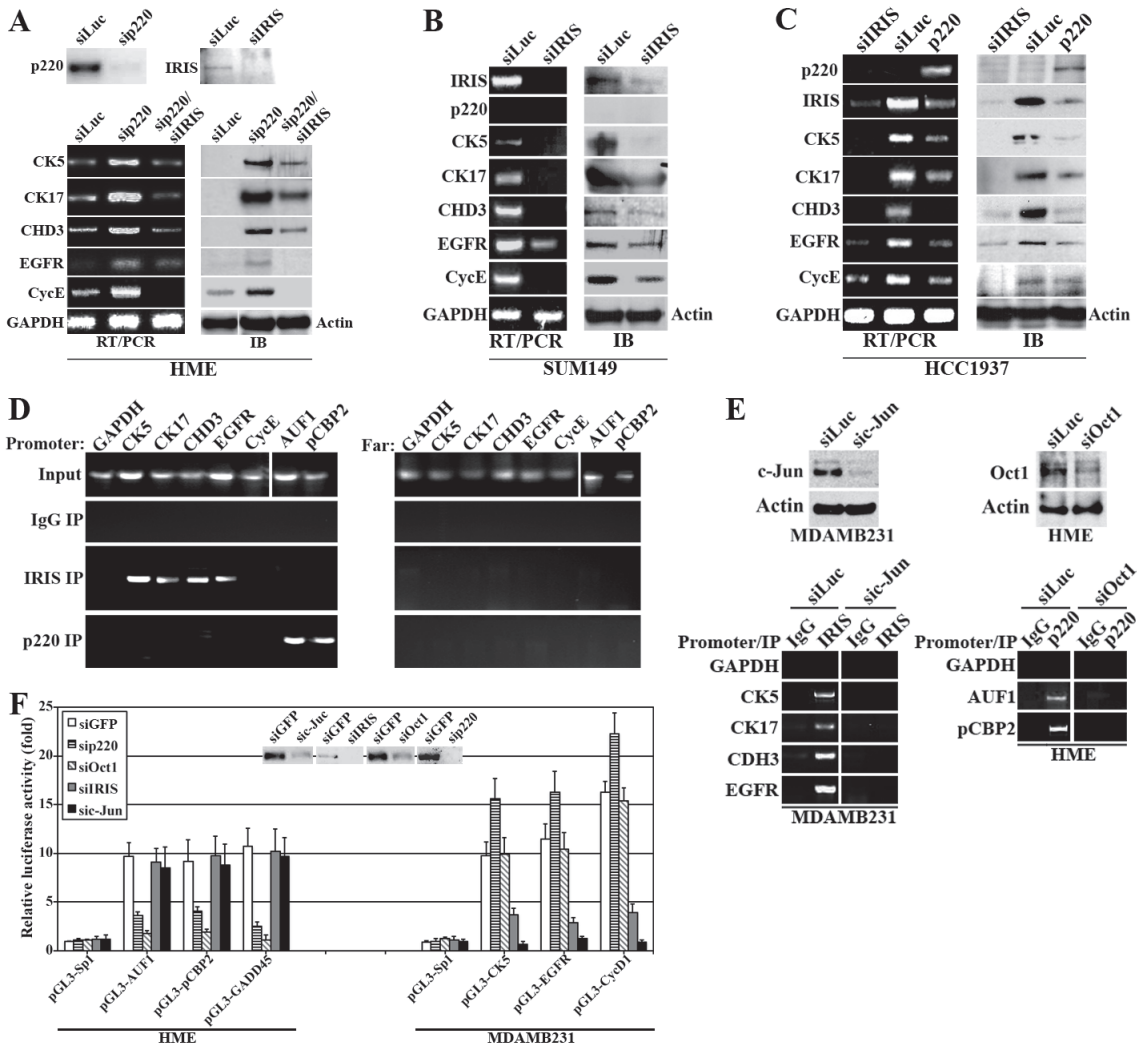
The effect of BRCA1-IRIS and BRCA1/p220 on the expression of several TN/BL markers Expression of the indicated TN/BL markers mRNAs (left) or proteins (right) in BRCA1/p220-silenced or BRCA1/p220 and BRCA1-IRIS co-silenced HME cells (A), BRCA1-IRIS-silenced SUM149 cells (B), BRCA1/p220 overexpressing or BRCA1-IRIS silenced HCC1937 cells (C). (D) PCR analysis showing promoters (left) or 10kb upstream regions (right) of CK5, CK17, CDH3, EGFR, cyclin E, AUF1 and pCBP2 in BRCA1-IRIS or BRCA1/p220 immunoprecipitation from cross-linked HME cells using mono-specific antibodies. (E) ChIP analysis of the promoters of the indicated TN/BL markers in control- or c-Jun-silenced MDAMB231 cells or control- or Oct1-silenced HME cells. (F) Analysis of the indicated promoters activation in HME cells (left) or MDAMB231 (right) depleted from BRCA1/p220, Oct1, BRCA1-IRIS or c-Jun. Inset is the effect of each siRNA on its cognate protein in HME cells.

The fact that BRCA1/p220-associated and TN/BL breast cancers commonly show early onset and aggressive diseases expressing no-/low-BRCA1/p220, made us wonder whether BRCA1/p220 loss enhances the TN/BL phenotype via upregulating BRCA1-IRIS. To test this hypothesis, BRCA1/p220 was silenced in the BRCA1/p220-high-/BRCA1-IRIS-low expressing HME cells (Figure 5A, upper panels). Total RNAs and proteins collected from these cells were then probed for the expression of the TN/BL markers, CK5, CK17, CDH3, EGFR and cyclin E [35]. As expected, BRCA1/p220 silencing upregulated the expression of the mRNAs and proteins of these markers (which otherwise expressed at low levels in HME cells, see Figure 5A, lower panels). More importantly, BRCA1-IRIS co-silencing in these cells decreased the expression of these markers (Figure 5A, lower panels).

Furthermore, BRCA1-IRIS silencing in the TN/BL cell line, SUM149 that expresses high levels of BRCA1-IRIS, CK5, CK17, CDH3, EGFR, cyclin E but no BRCA1/p220 (Figure 5B) significantly decreased the expression of these TN/BL markers mRNAs and proteins (Figure 5B). Whereas, HCC1937, the BRCA1/p220 mutant cell line that expresses high levels of BRCA1-IRIS and TN/BL markers, but no wild type BRCA1/p220 (Figure 5C), reconstitution with full-length BRCA1/p220 cDNA or silencing BRCA1-IRIS in them (Figure 5C) decreased the levels of TN/BL markers and BRCA1-IRIS mRNA and protein (Figure 5C). These data establish that BRCA1/p220 loss of expression or function enhances the TN/BL phenotype in breast cancer cells via upregulating BRCA1-IRIS expression.

### BRCA1/p220 binds AUF1 and pCBP2 promoters while BRCA1-IRIS binds CK5, CK17, CDH3, EGFR and cyclin E promoters

Next, we asked whether these effects are transcriptional. In chromatin immunoprecipitation (ChIP) experiments, exponentially growing HME cells were cross-linked, sonicated (to generate ~500bp DNA fragments), then extracts were immunoprecipitated with IgG (negative control), BRCA1-IRIS or BRCA1/p220 monoclonal antibodies. After Immunoprecipitation, cross-linking was reversed and PCR was used to search for specific DNA fragments immunoprecipitated with these antibodies. BRCA1-IRIS co-immunoprecipitated CK5, CK17, CDH3 and EGFR promoter fragments (Figure 5D, left), whereas BRCA1/p220 co-immunoprecipitated AUF1 and pCBP2 promoter fragments (Figure 5D, left). All promoters were present in the input of each experiment (Figure 5D, left), and while inputs also contained the fragments located ~10kb upstream of each promoter (Figure 5D, right), these fragments were not co-immunoprecipitated by BRCA1-IRIS or BRCA1/p220 (Figure 5D, right) antibodies. These data show that BRCA1/p220 binds AUF1 and pCBP2, while BRCA1-IRIS binds the promoters of several TN/BL markers.

### Potential transcriptional links necessary for BRCA1/p220 or BRCA1-IRIS

Using *in silico* analysis we compared AUF1 and pCBP2 (~1000bp upstream of +1 position) promoters in search for common factors that could link BRCA1/p220 to these genes transcription. An Oct1 binding site was common between AUF1 and pCBP2 promoters, which is interesting, since BRCA1/p220 was shown recently to induce transcription of several genes, including GADD45 through binding to Oct1 [37]. Also using *in silico* analysis we compared the promoter regions of these TN/BL genes (~1000bp upstream of +1 position) for common transcription binding sites, and found that all but cyclin E share an AP1 binding site. Importantly, we recently showed that BRCA1-IRIS induces cyclin D1 expression through binding to c-Jun/AP1 [32].

To study that, c-Jun was silenced in the BRCA1-IRIS-overexpressing MDAMB231 cell line (72h, Figure 5E, left upper panels) and Oct1 was silenced in the BRCA1/p220-expression HME cell line (72h, Figure 5E, right upper panels). ChIP analysis of extracts isolated from these cells confirmed that c-Jun-silencing in MDAMB231 cells significantly reduced the amounts of CK5, CK17, CH3 and EGFR promoter fragments co-immunoprecipitated by BRCA1-IRIS antibody (Figure 5E, left lower panels), and Oct1-silencing in HME cells, dramatically reduced the amount of AUF1 and pCBP2 promoter fragments co-immunoprecipitate by BRCA1/p220 antibody (Figure 5E, right lower panels).

To ascertain these relationships further, CK5, EGFR, AUF1, pCBP2 promoters were cloned upstream of the luciferase gene in the pGL3 reporter plasmid. Three Sp1 binding sites cloned upstream of the luciferase gene in this reporter plasmid was used as negative control. Positive controls for BRCA1-IRIS transcription activity was cyclin D1 promoter driving luciferase reporter (see [31]), and for BRCA1/p220 induced transcription activity was GADD45 driven luciferase reporter (see [37]). The pGL3-AUF1, -pCBP2 and -GADD45, -CK5, -EGFR and -CycD1 constructs were co-transfected with GFP-, BRCA1/p220-, Oct1-, BRCA1-IRIS- or c-Jun-silenced HME (inset in Figure 5F) or MDAMB231 (not shown) cells, respectively. Luciferase activity from each reporter following the different treatments was measured in each case 72h later.

As expected Oct1 silencing significantly decreased luciferase expression from GADD45, AUF1 and pCBP2 promoters (Figure 5F, left), but had no effect on CK5, EGFR or cyclin D1 promoters (Figure 5F, right). Importantly, BRCA1/p220 silencing also significantly suppressed luciferase expression from GADD45, AUF1 and pCBP2 promoters (Figure 5F, left), and as expected since BRCA1/p220 silencing upregulates BRCA1-IRIS expression, a slightly increased in luciferase expression from CK5, EGFR and cyclin D1 promoters was measured in these cells (Figure 5F, right). On the other hand, c-Jun silencing suppressed luciferase expression from cyclin D1, CK5 and EGFR promoters (Figure 5F, right) and more importantly, BRCA1-IRIS silencing also significantly reduced luciferase expression from these promoters (Figure 5F, right). Luciferase expression from AUF1, pCBP2 and GADD45 promoters was not affected by c-Jun or BRCA1-IRIS silencing (Figure 5F, left). These data show that BRCA1/p220 induces expression of AUF1 and pCBP2 through binding to and activating Oct1 on their promoters, whereas BRCA1-IRIS enhances expression of this subset of TN/BL genes by binding to and activating c-Jun on their promoters.

### Loss of BRCA1/p220 enhances tumor cell survival via BRCA1-IRIS overexpression

Next, we studied whether BRCA1-IRIS overexpression in no/low BRCA1/p220 expressing cells promotes their survival and hence drug resistance phenotype associated with TN/BL breast cancers. BRCA1-IRIS and BRCA1/p220 were separately or together silenced or BRCA1-IRIS was overexpressed alone or in BRCA1/p220-silenced (i.e. BRCA1/p220 silenced in induced IRISb cells) in HME cells. Sonicated proteins were analyzed for expression and activation of AKT and its down-stream targets survivin and BAD 72h later [38,39].

**Figure 6:**
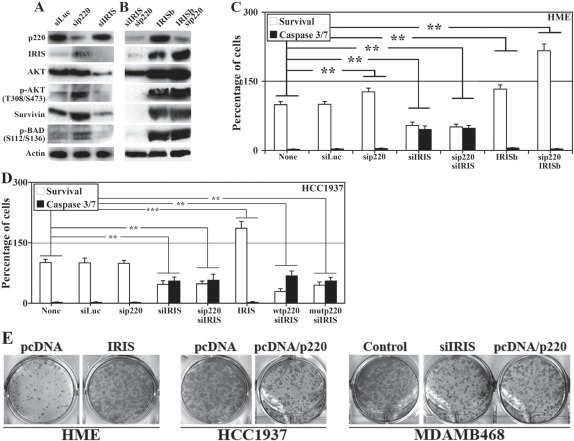
The effects of BRCA1-IRIS and/or BRCA1/p220 on the expression and activation of survival proteins, cell survival and transformation Expression or activation of the indicated proteins in control, BRCA1-IRIS, BRCA1/p220 silenced (A), BRCA1-IRIS overexpressing or BRCA1-IRIS overexpressing and BRCA1/p220-silenced (B) HME cells. (C) Percentage of viable (MTT assay analysis) or dying (caspase 3/7 assay analysis) cells, respectively in HME cells treated as in A and B. Data are presented as means ± SD from triplicate, done three independent times, whereas ** is a p≤0.01. (D) Analysis of viability detected using MTT assay and cell death detected using caspase 3/7 assays in BRCA1-IRIS-silenced and/or wild type or clinically relevant mutant BRCA1/p220 overexpressing HCC1937 cells. Data are presented as the means ± SD from triplicate, done three independent times, whereas ** is a p≤0.01. (E) Soft agar analysis of control or BRCA1-IRIS overexpressing HME cells (left), control or BRCA1/p220 overexpressing HCC1937 cells (middle), or control, BRCA1-IRIS-silenced or BRCA1/p22

Control treated HME cells express high levels of BRCA1/p220, AKT, survivin (Figure 6A) and low levels of BRCA1-IRIS, phosphorylated (on T308/S473)/activated AKT (hereafter p-AKT, Figure 6A) and phosphorylated (on S112/136)/inactivated BAD (hereafter p-BAD, Figure 6A). BRCA1-IRIS silencing had no effect on BRCA1/p220 expression (Figure 6A), dramatically decreased AKT, and survivin expression (Figure 6A, also [34]) and led to significant decrease in HME and HCC1937 cells viability as measured using MTT and activated caspase3/7, respectively (Figure 6C and D). BRCA1/p220 and BRCA1-IRIS co-silenced cells showed similar phenotypes to BRCA1-IRIS only silenced cells (Figure 6A-D).

Conversely, BRCA1/p220 silencing upregulated expression of BRCA1-IRIS, AKT, p-AKT, survivin and p-Bad (Figure 6A), which led to a slight but significant increase in viability and decrease in caspase3/7 activation (Figure 6C). BRCA1-IRIS overexpression (i.e. induced IRISb cells) had no effect on BRCA1/p220 expression, dramatically increased AKT, p-AKT, survivin (Figure 6B, also see [34]) and p-BAD expression (Figure 6B) and significantly increased HME and HCC1937 cells viability (Figure 6C and D). Indeed, BRCA1/p220 silencing in induced IRISb cells (which significantly increased BRCA1-IRIS, AKT, p-AKT, survivin and p-BAD levels, Figure 6B) or BRCA1-IRIS overexpression in HCC1937 dramatically reduced the level of activated caspase 3/7, which led to increase in cell number in both cell lines above control treated cells (Figure 6C and D). Importantly, overexpressing of wild type or clinically relevant BRCA1/p220 mutant in HCC1937 could not overcome the inhibitory effect observed with BRCA1-IRIS silencing (Figure 6D).

Finally, colony assay was used to assess the transformation capabilities of BRCA1-IRIS overexpression or BRCA1/p220 silencing. HME cells were transfected with control or BRCA1-IRIS-expressing vector, HCC1937 cells were transfected with control or BRCA1/p220-expressing vector, and MDAMB468 cells were transfected with appropriate control, BRCA1-IRIS siRNA or BRCA1/p220-expressing vector. All cell lines were grown in soft agar for 2 weeks, at which time formed colonies were stained and counted. Compared to control treated cells, BRCA1-IRIS overexpression increased the number of HME colonies (Figure 6E, left), BRCA1/p220 overexpression reduced the number of HCC1937 colonies (Figure 6E, middle) and BRCA1-IRIS silencing or BRCA1/p220 overexpression reduced MDAMB468 colonies (Figure 6E, right). These data show that BRCA1-IRIS overexpression, like BRCA1/p220 dowregulation initiates and/or maintains the transformation of mammary cells.

## DISCUSSION

BRCA1-IRIS is a novel BRCA1/p220 locus produced oncogene. BRCA1/p220 inhibition or BRCA1-IRIS overexpression in mammary epithelial cells enhances expression of cyclin D1, AKT and several other proliferation and survival proteins [31-34,40,41, and this study]. BRCA1/p220 loss [42] or BRCA1-IRIS overexpression (ElShamy, unpublished) confers tamoxifen resistance in mammary epithelial cells. These observations support the view that BRCA1-IRIS overexpressing cells are phenotypically equivalent to no/low BRCA1/p220 expressing cells. In this study we presented a mechanistical support for this notion. We showed that, at least partially, BRCA1/p220 loss increases BRCA1-IRIS expression by a post-transcriptional mechanism, explaining the inverse relationship between the two genes.

Human and rat BRCA1/p220 *mRNA* 3`-UTR (~1.5kb) contains several HuR binding sites [43]. The BRCA1-IRIS 3`-UTR (~300bp from intron 11), on the other hand contains binding sites for pCBP2 [22], AUF1 and HuR [21]. It is possible that HuR stabilizes both mRNAs, and that we fail to detect an effect of HuR on BRCA1-IRIS mRNA in HME cells is because the effect is perhaps masked by the fact that these cells are BRCA1/p220 proficient and hence express high levels of AUF1, that binds the same sites as HuR [21]. When BRCA1/p220 expression and/or function are lost and AUF1 expression is dropped (e.g., in cancer cells), BRCA1-IRIS *mRNA* is perhaps stabilized by HuR.

In fact, we recently performed immunohistochemical analysis of breast cancer tissue microarray (containing >300 aggressive breast cancer tumor samples) and discovered that compared to normal tissue, BRCA1-IRIS is overexpressed in the majority of these tumors, that expressed very low levels of AUF1 and pCBP2, high levels of cytoplasmic HuR and no BRCA1/p220 (ElShamy WM, unpublished data). Cytoplasmic HuR was proposed recently to be an independent prognostic factor for familial breast cancers and a poor prognosis factor for sporadic and familial breast cancers, or could even be a contributing factor to the disease [44,45]. In fact, in our recent publication [33] we showed a strong correlation between BRCA1-IRIS overexpression and cytoplasmic localization of HuR, *in vitro* [33].

pCBP2 binds to and activates BRCA1/p220 promoter [46]. It is possible that a positive feedback mechanism between BRCA1/p220 and pCBP2 exists, which is broken in *BRCA1/p220*-mutant or TN/BL cells. Further studies to elucidate the role of posttranscriptional mechanisms controlling BRCA1/p220 and BRCA1-IRIS mRNA expression in normal and breast cancer cells are required to elucidate the mechanism(s) underlying the development of familial and TN/BL breast cancers.

The inverse relationship we propose here between expression of BRCA1/p220 and BRCA1-IRIS seems not to be complete. For instance, although MCF7 and MDAMB231 cell lines express high levels of BRCA1-IRIS, both cell lines still express detectable levels of BRCA1/p220 (see above). It is possible that while intriguing, this mechanism is perhaps not the only mechanism involved. In this regard, we recently found that BRCA1-IRIS was overexpressed in xenograft or orthotopic tumors generated using HME cells overexpressing TERT/LT/Ras^V12^ [47]. Oncogenic Ras is known to suppress expression of several transcription factors, including vitamin D during mammary epithelial cell transformation [48]. It is possible that vitamin D is a transcription suppressor of BRCA1-IRIS. However, this remains only a hypothesis until the promoter of BRCA1-IRIS has been cloned.

Alternatively, oncogenic Ras overexpression was shown recently to decrease the expression of AUF1 during mammary cell transformation [49]. It is possible that in BRCA1/p220 expressing breast cancer tumor cells, Ras^V12^ instead stabilizes BRCA1-IRIS *mRNA* leading to its protein overexpression. However, what was even more surprising is the fact that tumors generated using HME cells overexpressing TERT/LT/BRCA1-IRIS were BRCA1/p220-negative [47]. If true also in human tumors, this suggests that BRCA1/p220 loss of expression increases BRCA1-IRIS, which in a negative feedback mechanism suppress BRCA1/p220 expression.

It is possible that patients with tumors lacking BRCA1/p220 expression or function are hit twice. Once by losing the powerful tumor suppressor, BRCA1/p220, which is involved in DNA-damage repair, cell cycle arrest, transcription and chromatin remodeling and a second time by gaining the powerful oncogene, BRCA1-IRIS, which enhances cell proliferation and survival when overexpressed. This combined effect, perhaps, contributes to increase aggressiveness and drug resistance phenotypes in no/low BRCA1/p220 expressing breast tumors, and support the view that the two proteins affect a linear pathway(s), in which BRCA1/p220 silencing and/or BRCA1-IRIS overexpression gives survival advantages to cancer cells and promotes the formation of death resistant TN/BL breast cancer cells. We therefore propose that chemotherapeutical targeting of BRCA1-IRIS might be beneficial in eradicating *BRCA1/p220*-associated or TN/BL tumors cancer diseases.

## MATERIALS AND METHODS

### Cell culture and transfection

Human mammary epithelial cells were cultured in MEGM modified medium (Lonza). All other breast cancer cell lines used in this study were grown in RPMI-1640 supplemented with 10% FBS. Small interfering RNA (siRNA) targeting BRCA1/p220 and BRCA1-IRIS were described earlier [30], whereas BARD1, AUF, pCPB2 and HuR siRNAs were from Dharmacon. Transfections of plasmids with or without siRNAs were done using oligofectamine (Invitrogen). Plasmid transfection was done using lipofectamine 2000 (Invitrogen). Cells were harvested after transfection at the indicated times.

### Establishment of doxycycline-induced BRCA1-IRIS expression in HME cells

Full-length BRCA1-IRIS cDNAs containing the entire 3`-UTR was amplified from HME total RNA using primers described earlier [30] was cloned in the pRevTRE plasmid (Clontech). pRev-TRE-IRIS was subsequently transfected into selected, rtTA-producing HME clones followed by selection with 150μg/ml hygromycin B (Sigma). Tet-responsive expression of BRCA1-IRIS by doxycycline (1-2 μg, Clontech) was monitored by using real time RT/PCR and/or by western analysis. The authenticity of all constructs used for transfections was verified by sequencing.

### Transient infection of BRCA1-IRIS or BRCA1/p220 cDNA

In some experiments a lentivirus expressing full-length BRCA1/p220 or BRCA1-IRIS was used to express either protein in transient expression. Verification of expression was done using western blotting.

#### Western blot analysis

Whole-cell lysates were prepared using cell sonication [33,34]. Protein lysates were resolved by NuPAGE gels (Invitrogen) and transferred onto nitrocellulose or PVDF membranes. Antibodies used to detect BRCA1-IRIS are mouse monoclonal antibody developed in the lab, to detect BRCA1/p220 we use the mouse monoclonal antibody SG-11 (Calbiochem, San Diego, Calif), AUF1, pCBP2, HuR and β-actin (Sigma), AKT1, AKT2, p-(T308/S473)-AKT, survivin and p-(S112/136)-BAD (Cell Signaling), rabbit monoclonal CK5 (ab75869), CK17 (ab51056), mouse monoclonal CDH3 (ab19350), EGFR (ab5368-13) and rabbit polyclonal cyclin E (ab93161) all from abcam. Following secondary antibody incubations, signals were visualized by enhanced chemo-luminescence.

### RT/PCR analysis

Total RNA, isolated with Trizol (Gibco, Life Technologies) and DNaseI-treated, was routinely used in RT-PCR experiments using SuperScript One-Step RT-PCR with Platinum Taq (Invitrogen). Routinely, 5 μg of total RNA or 10 μg of poly A+ RNA were used as a template in each reaction for amplification of ~450bp of *BRCA1-IRIS* RNA (that is, nucleotides 3,744–4,199 of *BRCA1-IRIS* cDNA), ~486bp of *BRCA1/p220* RNA (that is, nucleotides 4,674–5,160 of *BRCA1* cDNA) or ~350bp of *GAPDH* RNA, according to the manufacturer's instructions. Primers to amplify *CK5*, *CK17*, *CDH3*, *EGFR* and *cyclin E* mRNAs are shown in Supplemental Table 1.

### mRNA stability

For mRNA half-life assessments, three independent experiments were performed. Actinomycin D (5μg/ml) was added and total RNA was prepared at the times indicated; mRNA half-lives were calculated after quantifying by RT/qPCR, normalizing to 18S RNA levels (using a 1:20 dilution of the stock sample), plotting on logarithmic scales using GraphPad Prism, and calculating the time period required for a given transcript to undergo a reduction to one-half of its initial abundance (at time zero, before adding actinomycin D) using non-linear regression analysis. Comparisons of treatment outcomes were tested for statistical differences using the Student t-test for paired data. Statistical significance was assumed at a *p*-value of ≤ 0.05.

### Immunoprecipitation of RNP complexes

Immunoprecipitation (IP) of AUF1, pCBP2 or HuR and *BRCA1-IRIS mRNA* complexes from HME cell lysates was used to assess the association of the endogenous proteins with endogenous *BRCA1-IRIS* mRNA. The IP assay was performed essentially as described earlier [33]. Following Immunoprecipitation, extensive washes and digestion of proteins in the IP material [33], the RNA was extracted and used to perform reverse transcription (RT) followed by PCR to detect the presence of *BRCA1-IRIS* mRNA using gene-specific primer pairs described in [30]. GAPDH mRNA was used to normalize the data. We routinely normalized the results by measuring in parallel the binding of *BRCA1-IRIS* mRNA to IgG and to anti-AUF1, pCBP2 or HuR antibodies [33].

### Luciferase analysis

A 210bp representing the entire 3`-UTR of *BRCA1-IRIS* or the 3`-UTR of *c-fos* [30,50,51] were subcloned into the *Hpa*I site of the Rous sarcoma virus-luciferase (RSV-Luc) expression vector. A plasmid encoding RSV-β-galactosidase (RSV-β-Gal; ATCC) was co-transfected as an internal control. In other experiments, fragments (~1-3kb) containing human *AUF1, pCBP2, CK5, EGFR, CycD1* or *GADD45* genes promoter elements were generated by PCR amplification from human genomic DNA (G304A, Promega). Each promoter fragment was cloned upstream of the firefly luciferase reporter gene in the *pGL3-Basic* vector (Promega) using the protocol described in Chock et al., 2010a. The resultant plasmids were designated as *pGL3-AUF1, -pCBP2, -GADD45, -CK5, -EGFR and –CycD1*. Comparisons of treatment outcomes were tested for statistical differences using the Student t-test for paired data. Statistical significance was assumed at a P-value of ≤ 0.05.

### Active Caspase 3/7 detection and MTS assays

The Apo-ONE® Homogeneous Caspase-3/7 kit and CellTiter 96® Aqueous Non-Radioactive Cell Proliferation Assay kit were used according to supplier (Promega) protocol. Comparisons of treatment outcomes were tested for statistical differences using the Student t-test for paired data. Statistical significance was assumed at a *p*-value of ≤ 0.05.

### Chromatin immunoprecipitation (ChIP) analysis

ChIP was performed as described in [30]. PCR conditions are as follows; 10min at 94°C to activate the Taq polymerase followed by 30 cycles of denaturation for 1min at 94°C, annealing for 1min at 60°C, elongation for 1min at 72°C and a final extension for 7min at 72°C. Primers to amplify the immediate promoter regions or a regions located ~10kb upstream of *CK5*, *CK17*, *CDH3*, *EGFR* and *cyclin E* are shown in Supplemental Table 1.

## SUPPLEMENTARY TABLE


